# Marine Fungi Select and Transport Aerobic and Anaerobic Bacterial Populations from Polycyclic Aromatic Hydrocarbon-Contaminated Sediments

**DOI:** 10.1128/mbio.02761-22

**Published:** 2023-02-14

**Authors:** Joyce Álvarez-Barragán, Cristiana Cravo-Laureau, Bijing Xiong, Lukas Y. Wick, Robert Duran

**Affiliations:** a Universite de Pau et des Pays de l’Adour, E2S UPPA, CNRS, IPREM, Pau, France; b Helmholtz Centre for Environmental Research–UFZ, Department of Environmental Microbiology, Leipzig, Germany; Oregon State University

**Keywords:** fungal translocation network, fungal-bacterial interactions, hyphal selection, oxygen consumption, bacterial diversity, bacteria dispersion

## Abstract

The organization of microbial communities in marine sediment relies on complex biotic and abiotic interactions. Among them, the interaction between fungi and bacteria plays a crucial role building specific microbial assemblages, resulting in metabolic networks adapted to environmental conditions. The fungal-bacterial interaction (FBI) includes bacterial translocation via fungal mycelia, allowing bacterial dispersion, and ecological niche colonization. In order to demonstrate that the translocation of bacteria through fungal mycelia involves bacterial selection, the mycelia of two fungi isolated from marine coastal sediment, Alternaria destruens F10.81 and Fusarium pseudonygamai F5.76, showing different strategies for uptake of polycyclic aromatic hydrocarbon (PAH), homogenous internalization and vacuole forming respectively, were used to translocate bacteria through hydrophobic hydrocarbon contaminated sediments. *A. destruens* F10.81 selected four specific bacteria, while bacterial selection by *F. pseudonygamai* F5.76 was not evident. Among the bacteria selected by *A. destruens* F10.81, Spirochaeta litoralis, known as strictly anaerobic bacterium, was identified, indicating that *A. destruens* F10.81 selects and transports both aerobic and anaerobic bacteria. Such a result is consistent with the observed formation of anoxic micro-niches in areas surrounding and affected by fungal hyphae. Our findings provide new insights on the selection and dispersion of bacterial communities by fungi, which are crucial for the organization of microbial communities and their functioning in coastal PAH-contaminated sediments.

## INTRODUCTION

Bacteria coexist with fungi as free-living organisms or in the fungal intracellular environment (obligate endobacteria) or both (facultative endobacteria) (1). The number of bacteria able to establish symbiotic interactions with fungi is limited (1–6), indicating that fungal-bacterial interactions (FBI) involve selection mechanisms. Bacteria associated directly with the fungal hyphae (2, 7) living in the hyphosphere, the zone influenced by the hyphae (1), have been shown to be essential for biogeochemical processes such as the depletion of polycyclic aromatic hydrocarbons (PAHs), their hydrocarbon degradation capacities being complementary to the fungal activities to achieve the complete mineralization (8). Indeed, the bacteria colonizing the hyphosphere have been shown able to degrade the metabolites produced by fungi during the first steps of PAH degradation (9, 10).

PAHs are hydrophobic compounds of major concern for the environment because they are toxic and resilient (11). PAHs, resulting from fossil fuel-based human activities, enter into aquatic ecosystems in different ways including direct inputs of crude oil, runoff of urban and industrial wastewaters, and atmospheric deposition of oil-combustion emissions (11). They accumulate in marine sediments, where they directly affect benthic organisms, then threatening the food chain (12, 13). Biotic and abiotic factors determine the fate of PAHs in marine sediments (11). A large diversity of hydrocarbon-degrading microorganisms has been described (11, 14), revealing the PAH degradation mechanisms (aerobic and anaerobic), which are well known for bacteria and fungi (11, 15, 16). Microorganisms and their interactions play a key role in aerobic and anaerobic PAH degradation (8, 11). Among these interactions, fungal-bacterial interactions (FBI) have been demonstrated to stimulate PAH degradation (17). The FBI in the hyphosphere has been demonstrated to be synergistic (complementary) and mutualistic, where both partners get benefits for their development (1). Bacteria influence the metabolism and physiology of fungi (18–20), while fungal hyphae transfer nutrients, water, and carbon sources to bacteria (21–25). In addition, fungal mycelium has been demonstrated to be used as translocation network (“fungal highway”) for the dispersion of bacteria (5, 10, 26), including bacteria able to degrade PAHs such as naphthalene, phenanthrene, or pyrene (10, 27). By random or chemotaxis-driven mechanisms (28) they allow bacteria to colonize new environmental niches, to cross obstacles (29, 30), or even to carry along phages (31). Dispersal thereby is based on bacterial swimming or surface-associated motility, although transport by growing hyphal structures may not be excluded (32). Translocation networks have been largely studied in soils but no attention has been paid to translocation networks in marine sediments because it is assumed that the translocation of bacteria is not limited in aquatic environments (33). Nevertheless, most microorganisms are rarely living freely as a single-species population but, rather, have a lifestyle involving microbial interactions in many ecosystems, including coastal sediment (34). The marine sediments are composed of heterogeneous particles that can go from microscopic clays to large boulders (35) and present diverse physical-chemical conditions influencing bacterial dispersal and biogeochemical processes, especially those involving PAHs. For example, salinity reduces PAH solubility, resulting in aggregates that accumulate in sediments (36, 37). The PAHs entrapped in the sediment matrix create hydrophobic patches (38), restricting bacterial dispersion and limiting their degradation because of oxygen limitation (39). It has been demonstrated that fungi extend their mycelia through marine sediments, providing space and support for bacterial dispersion (40), which can provide access to PAHs as demonstrated for soil (41). Because marine sediments host a particular fungal diversity (42–44), it is expected that specific FBI are established in sediments, allowing obstacles to be overcome and favoring accessibility to PAHs. From an ecological point of view, the selection and dispersion of bacterial communities by fungi are crucial for the organization of microbial communities and their functioning in coastal PAH-contaminated sediments.

Recently, network analysis revealed the cooccurrence of specific bacteria with fungi in coastal sediment, suggesting that FBI might play a crucial role in the marine environment (45). Furthermore, the presence of PAH altered the network, revealing that the abundance of specific bacteria was strongly correlated with the abundance of specific fungi (45). Such strong correlations, indicating the specific cooccurrence of bacteria and fungi, support the hypothesis that bacteria and fungi can establish specific interactions through biotic associations. It has been shown that fungi develop different strategies to take up PAH (46), which depends on cell surface properties (47). It is likely that the specificity of FBI also depends on fungal cell surface properties, especially in the presence of hydrophobic compounds such as PAH.

We hypothesize that the translocation of bacteria through fungal mycelia involves bacterial selection resulting in a specific FBI. In order to test this hypothesis, microbial communities from coastal sediments were characterized along the fungal mycelia during translocation through hydrophobic patches under PAH-contaminated conditions. The microbial communities selected by the mycelia of two fungi, isolated from marine coastal sediments that were chosen because they exhibit different membrane properties since they adopt distinct PAH uptake strategies, were compared, aiming to demonstrate the specificity of the FBI.

## RESULTS AND DISCUSSION

### Validation of the experimental setup.

**FIG 1:**
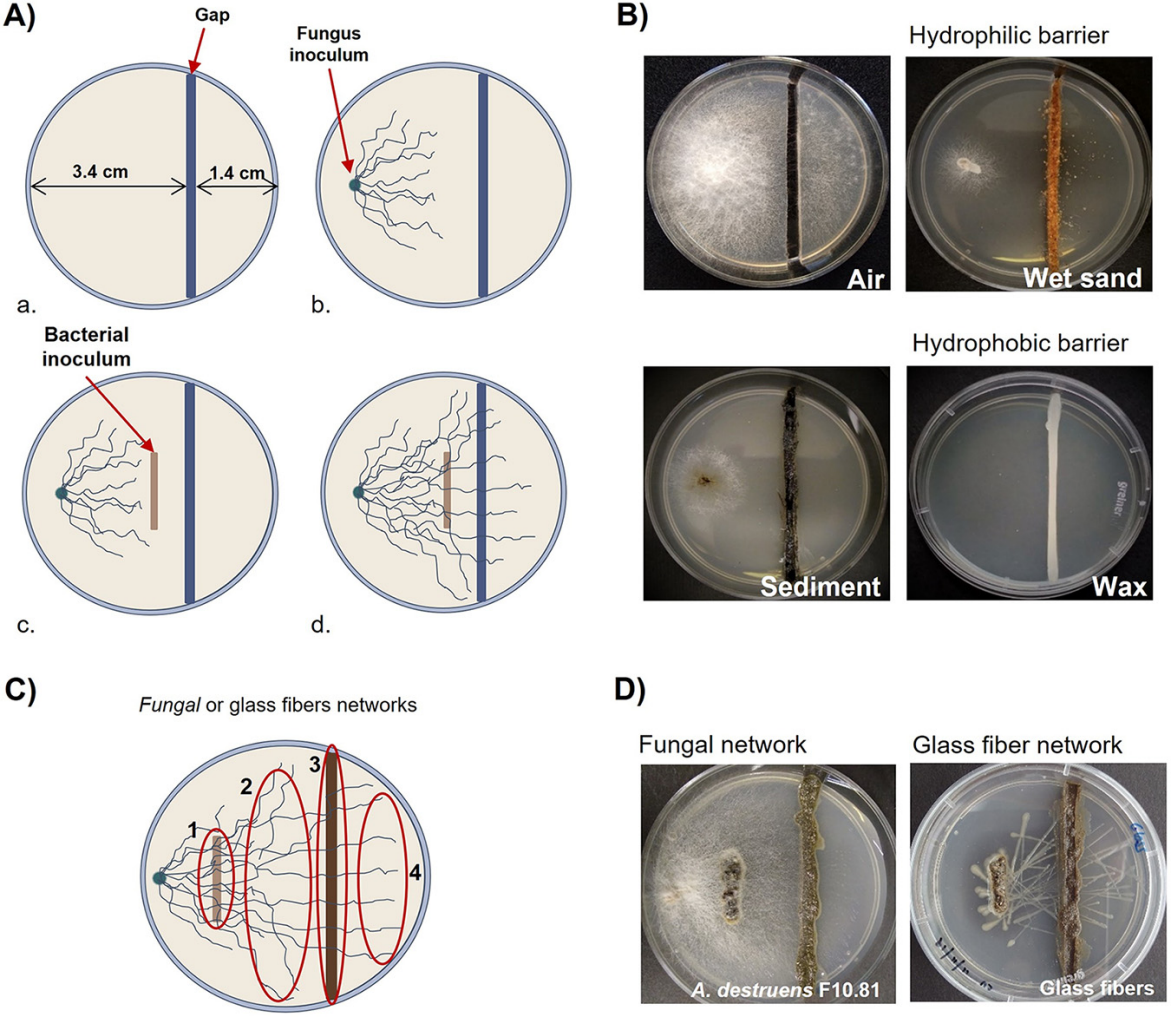
(A to D) Experimental design for dispersal of bacterial isolates (A and B) and bacterial communities (C and D) along mycelial networks. (A) For isolated bacteria translocated over physical barriers (air, wet sand, sediment, and wax) (a) before inoculation, (b) 48 h after fungus inoculation, (c) at the bacterial inoculation, (d) and 10 days after bacterial inoculation, the presence of bacteria was observed after the gap. (B) Pictures showing the different gaps used. (C) Bacterial community transport through fungal or glass fibers translocation networks. The sampling points are indicated: sediment inoculum (1), before the gap (2), sediment gap (3), and after the gap (4). (D) Pictures showing the experimental device after incubation in the presence of fungi and glass fibers.

In order to confirm the fungal capacity to translocate bacteria through the different physical barriers in the presence of PAH, eight bacteria isolated from PAH-contaminated coastal sediments were tested for their ability to cross the gaps thanks to fungal mycelia (Fig. 1). The bacterial dispersal experiments were performed in petri dishes in which solid medium was separated in 2 compartments by a gap (Fig. 1A-a). The gaps were filled with different hydrophobic or hydrophilic matrixes to mimic spatially distinct barriers (Fig. 1B). Because PAH molecules are toxic for bacterial growth, the seven bacteria able to grow in the presence of pyrene (PAH model molecule) were tested for translocation, while *Bacillus* sp. strain B2, the negative control sensitive to pyrene, was not translocated (Table 1). Two fungi, Alternaria destruens F10.81 and Fusarium pseudonygamai F5.76, isolated from hydrocarbon-contaminated marine coastal sediments (42), were used as translocation networks for their ability to grow in the presence of PAH. The fungi were selected because they likely differ in their cell surface properties since they exhibited different PAH uptake strategies (42), which rely on cell surface properties such as hydrophobicity (47). PAH uptake by *A. destruens* F10.81 involves homogenous internalization, while *F. pseudonygamai* F5.76 forms PAH vacuoles (42). Thus, the comparison of the bacterial translocation capacities of both fungi will inform on the specific bacterial selection by fungi during translocation. Despite their difference, both fungi were able to translocate bacteria along the mycelia in the presence of pyrene. However, some bacteria showed different behaviors according to the fungi and the nature of the gap, obviously illustrated by an *Exiguobacterium* sp. that was able to be translocated through the hydrophilic gap by *A. destruens* F10.58, while it was translocated through hydrophobic gaps by *F. pseudonygamai* F5.76 (Table 1). Such an observation was consistent with the fact that the interaction between bacteria and fungi might also depend on environmental factors (48–50).

**TABLE 1:** Bacterial characteristics and their translocation capacities through the hyphae of *F. pseudonygamai* F5.76 (F) and *A. destruens* F10.58 (A)

Strain number	Bacterium	Bacterial motility and growth in PAHs			Translocation through gap^*c*^^,^^*d*^											
		Growth on pyrene^*b*^	Swimming^*a*^ (cm/ 16 h)	Swarming^*a*^	Air gap			Wet sand gap			Sediment gap			Wax gap		
				(cm/16 h)	C	F	A	C	F	A	C	F	A	C	F	A
B1	Pseudomonas sp.	+	3.5 ± 0.4	7.4 ± 1.2	+	−	−	+	+	+	−	+	+	−	+	+
B2	*Bacillus* sp.	−	2.8 ± 0.3		−	−	−	−	−	−	−	−	−	−	−	−
B3	*Bacillus* sp.	+ +			−	−	−	−	−	−	−	−	−	−	−	−
B4	Vibrio anguillarum	+ +	3.8 ± 0.2		−	−	−	+	−	+	−	+	+	−	+	+
B5	Vibrio anguillarum	+ +	3.5 ± 0.3		−	+	+	+	+	+	−	+	+	−	+	+
B6	Pseudomonas sp.	+	4.1 ± 0.2	8.1 ± 0.1	+	−	+	+	+	+	−	+	+	−	+	+
B7	*Bacillus* sp.	+ +	4.7 ± 0.3	2.8 ± 0.8	+	−	+	+	+	+	−	+	+	−	+	+
B8	*Exiguobacterium* sp.	+ +	1.9 ± 0.2		−	−	−	+	−	+	−	+	−	−	+	−

a Cultured along 16 h at 25°C in swimming and swarming media.

b Seawater minimal media containing 30 mg L^−1^ of pyrene as unique carbon source.

c swMM containing 10% LB and 30 mg L^−1^ pyrene.

d C, control: no translocation network; F, *F. pseudonygamai* F5.76; A, *A. destruens* F10.58 −, no growth; +, growth. For columns 6 to 17: −, no translocation; +, translocation.

Only the pyrene-tolerant bacteria with motility (swimming or swarming) were translocated (Table 1). Among them, all bacteria exhibiting swimming motility were able to cross the hydrophilic (wet sand) gap even in the abiotic control (glass fiber) (Table 1). Only bacteria exhibiting swarming motility were able to cross the nonpolar fluid (air) gap in the abiotic control, but the presence of fungi hindered Pseudomonas sp. B1 to cross it while allowing the swimming Vibrio anguillarum B5 to cross the air gap (Table 1). However, crossing hydrophobic gaps (hydrocarbon-contaminated sediments and wax) was effective only with a fungal translocation network (Table 1). These results showed that motility is a crucial characteristic of bacteria for transport along the fungal translocation networks, as previously reported for isolated bacteria (10). It is likely that the motility allows bacterial movement along the water film on the hyphal surface (50). It has been suggested that the motility through fungal hyphae (fungal highway) provides an advantage in the soil environment as the fungal hyphae guide bacteria to arrive more efficiently to organic and nutrient hot spots (50). Although translocation networks in marine sediments have been neglected, because it is assumed that the translocation of bacteria is not limited in aquatic environments (33), fungal highways are likely to play a similar role in marine sediments, guiding bacteria for colonizing microniches. The isolated coastal bacteria are thus able to cross hydrophobic gaps through a translocation network constituted by hyphae of coastal fungi. This preliminary result allows us to assess the selectivity of bacterial communities from coastal sediments by fungal hypha.

### Bacterial selection by fungal translocation network.

In order to determine the specificity of the bacteria transported by the fungal hypha, the petri dishes for analyzing the transport of bacteria from bacterial communities of PAH-contaminated sediments were inoculated with two different sediments from hydrocarbon-polluted areas on the French Mediterranean coast. Although bacterial communities adapted to the presence of hydrocarbons inhabit both sediments, it is expected that they present different bacterial compositions because they are from environments with distinct salinity, Etang de Berre being brackish (15 to 25 practical salinity unit [PSU]) and Canal Vieil being marine (35 to 37 PSU) (16, 51).

The bacterial communities were characterized at different sampling points along the translocation networks crossing a hydrocarbon-contaminated hydrophobic sediment gap (Fig. 1C). The bacterial communities collected on the sampling points along the fungal translocation networks (*A. destruens* F10.81 and *F. pseudonygamai* F5.76) were compared to those obtained with a glass fiber translocation network control. The sequencing of the bacterial communities provided 2,985,157 reads of high-quality sequences; after trimming, the obtained 1,699,056 sequences were distributed within 1,882 amplicon sequence variants (ASVs; Table 2).

**TABLE 2:** Alpha diversity indices of the translocated bacterial communities obtained at the different sampling points transported along different translocation networks^*a*^

Source	Index	Sediment cultivable control^*b*^	*A. destruens* F10.58				*F. pseudonygamai* F5.76				Glass fiber			
		Inoculum	Inoculum^*c*^	Before^*c*^	Gap^*c*^	After^*c*^	Inoculum^*c*^	Before^*c*^	Gap^*c*^	After^*c*^	Inoculum^*c*^	Before^*c*^	Gap^*c*^	After^*b*^
Canal Vieil	Reads (*n*)	37,147 ± 3,504	27,007 ± 2,796	41,234 ± 3,659	35,335 ± 1,367	37,752 ± 7,157	16,819 ± 12,089	32,294 ± 3,416	33,735 ± 4,715	38,393 ± 5,366	35,395 ± 5,536	35,264 ± 7,549	32,790 ± 1,520	38 183 ± 3 282
	Trimmed sequences (*n*)	16,415 ± 1,442	13,563 ± 2,287	24,005 ± 2,965	21,142 ± 941	23,301 ± 3,487	6,295 ± 5,035	16,605 ± 3,743	18,554 ± 3,233	27,196 ± 2,457	17,680 ± 4,013	22,502 ± 4,570	18,459 ± 858	23 127 ± 3 501
	Richness (*R*)^*d*^	116 ± 43.49	102.33 ± 14.04	26 ± 15.87	31.33 ± 8.5	35 ± 20.42	81.67 ± 51.29	41.33 ± 4.51	43.67 ± 35.8	17.67 ± 8.96	114.67 ± 23.12	48.67 ± 13.5	41.67 ± 8.96	42.33 ± 24.21
	Shannon (*H*)	2.86 ± 0.42	2.82 ± 0.35	1.15 ± 0.66	1.54 ± 0.32	1.46 ± 0.31	3.32 ± 0.44	1.43 ± 0.5	1.35 ± 0.13	0.22 ± 0.2	2.76 ± 0.49	1.09 ± 0.22	1.56 ± 0.38	1.62 ± 0.93
Etang de Berre	Reads	40,045 ± 5,648	34,298 ± 9,765	43,494 ± 1,679	45,395 ± 4,033	43,691 ± 17,164	35,170 ± 8,974	39,552 ± 10,649	38,288 ± 7,972	32,874 ± 8,318	33,662 ± 10,307	36,848 ± 5,798	31,662 ± 8,488	33 606 ± 5 086
	Trimmed sequences	18,188 ± 3,109	14,807 ± 4,102	32,020 ± 1,798	34,536 ± 1,447	26,942 ± 2,904	13,780 ± 2,564	23,353 ± 6,129	22 177 ± 4,660	24,757 ± 7,804	15,703 ± 5,506	24,347 ± 6,580	20,121 ± 4,259	25 040 ± 5 066
	Richness (*R*)^*d*^	110.33 ± 54.63	109 ± 25.24	28.67 ± 9.61	6.67 ± 5.51	25.67 ± 22.18	129 ± 12.29	64.67 ± 72.28	27.67 ± 4.51	36.67 ± 37.54	74.33 ± 11.37	33.33 ± 14.47	52.67 ± 50.58	38.33 ± 25.11
	Shannon (*H*)	2.66 ± 0.27	3.14 ± 0.07	0.7 ± 0.21	0.18 ± 0.09	0.23 ± 0.17	3.42 ± 0.05	1.36 ± 0.92	1.31 ± 0.2	0.43 ± 0.48	2.43 ± 0.32	1.32 ± 0.5	1.05 ± 0.83	0.46 ± 0.24

a All analyses were performed in triplicate.

b Sediment cultivable control corresponds to the sediment cultivable control after 5 days of incubation at 25°C in seawater minimal medium containing 10% LB and 30 mg L^−1^ pyrene.

c Sampling points in the fungal translocation network setup.

d ASV richness.

The ASV richness (*R*) of the bacterial communities collected along the translocation networks followed similar trends irrespective of both the network and the origin of the sediment, being higher at the inoculum point than at the other sampling points along the translocation network (Table 2). Such a result suggested that the translocation network selects bacteria, likely the motile bacteria (10) probably exhibiting other specific characteristics. However, regarding the bacterial diversity Shannon index (*H*), different trends were observed according the origin of the sediment (Table 2). When comparing the bacterial communities of the inoculum point with the control, corresponding to the cultivable bacterial community from the sediments, differences were observed according to the origin of sediments. For the Canal Vieil sediment, the inoculum showed similar Shannon indexes (*H*) irrespective of the presence or absence of a translocation network. In contrast, for Etang de Berre sediment, the Shannon index (*H*) of the inoculum was significantly higher (*t* test, *P* < 0.05) for *F. pseudonygamai* F5.76 and for *A. destruens* F10.81, while it was significantly lower (*t* test, *P* < 0.05) for glass fibers (Table 2).

It is likely that the presence of translocation networks had different effects according to the origin of the microbial community. Indeed, previous studies have shown that the microbial community composition is dependent on physical-chemical parameters (52), which probably affect the bacterial population able to be translocated. Also, the differences observed on the Shannon index (*H*) with the bacterial community from Etang de Berre between fungal network and glass fiber network suggested that the presence of fungi affect bacterial growth (19), favoring or inhibiting the growth of bacteria, resulting in bacterial selection. The bacterial selection includes direct mechanisms, the fungi producing either metabolites that favor the bacterial growth (53) or antibiotics that inhibit bacteria (19). Indirect mechanisms, such as modification of pH (54) or of oxygen availability (55), have also been described as selection mechanisms.

Regarding the modification of bacterial diversity along the translocation network, significant modification of the Shannon index (*H*) was observed only with bacterial communities from the Etang de Berre sediment (Table 2). The Shannon index (*H*) decreased along the translocation network of both fungi, being significantly different (*t* test, *P* < 0.05) along the sampling points of the *A. destruens* F10.81 translocation network and significantly lower (*t* test, *P* < 0.05) in the gap and after the gap sampling points of the *F. pseudonygamai* F5.76 translocation network (Table 2). Such observation further supports that a bacterial selection occurs along the translocation network. In contrast, for the glass fiber translocation network, the Shannon index (*H*) was significantly lower (*t* test, *P* < 0.05) only for after the gap with Etang de Berre sediment. Thus, the nature of the translocation network affects the diversity of microbial communities along the sampling points, suggesting a selection of bacterial communities related to fungal-bacterial interaction. Consistently, several studies have demonstrated fungal-bacterial interactions (FBI) involving complex molecular mechanisms (56, 57), which might result in bacterial community selection (57). In order to characterize the FBI and demonstrate bacterial selection, the bacterial community composition and structure were further analyzed.

**FIG 2:**
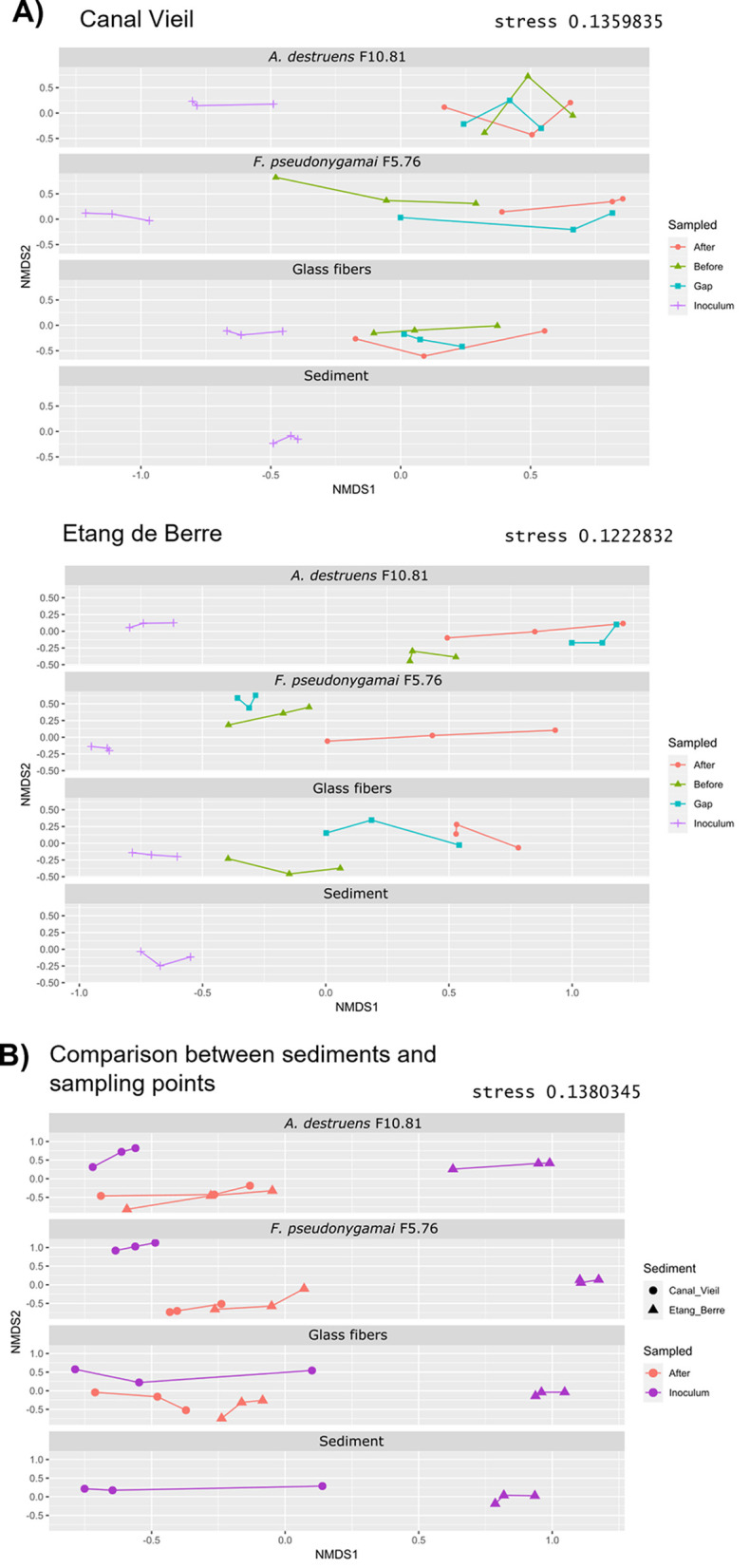
Comparison of bacterial communities by nonmetric multidimensional scaling (NMDS). The NMDSs, based on Bray-Curtis pairwise dissimilarity, were performed including all samples and then dispatched according to the translocation networks for representation. (A) Comparison of bacterial communities from the different sampling points in each translocation network with sediments from Canal Vieil and Etang de Berre, and in sediment cultivable control without a translocation network (sediment). (B) Comparison of bacterial communities obtained from Canal Vieil and Etang de Berre sediments in each translocation network at inoculum and after the gap sampling points and in sediment cultivable control without a translocation network (sediment).

The bacterial communities of the inoculum, from both the Canal Vieil and Etang de Berre sediments, were different from those collected along the sampling points (before the gap, in the gap, and after the gap) on the translocation networks (Fig. 2A). For Canal Vieil, the only significant difference (permutational multivariate analysis of variance [PERMANOVA], *P* < 0.05) was observed between bacterial communities before and after the gap on the *F. pseudonygamai* F5.76 translocation network (Fig. 2A). In contrast, for Etang de Berre, bacterial communities were not different before and after the gap for fungal translocation networks (PERMANOVA, *P* > 0.05), while they were different for the glass fiber translocation network (PERMANOVA, *P* < 0.05) (Fig. 2A). Consistent with alpha diversity analysis, it is likely that the selection of bacteria was dependent on the origin of the microbial community and the translocation network.

The inoculum compositions of bacterial communities from the Etang de Berre and Canal Vieil sediments were different (PERMANOVA, *P* < 0.001) (Fig. 2B). Despite the fact that the bacterial communities of the inoculum from Etang de Berre and Canal Vieil were different irrespective of the translocation network, the bacterial communities were similar after the gap with the fungal translocation networks for both sediments (*A. destruens* F10.81, *F. pseudonygamai* F5.76; PERMANOVA, *P* > 0.05) (Fig. 2B). Such an observation further supports the selection of bacterial communities by the fungal translocation network, which was consistent with the study showing that fungal networks shape bacterial community structures (57).

**FIG 3:**
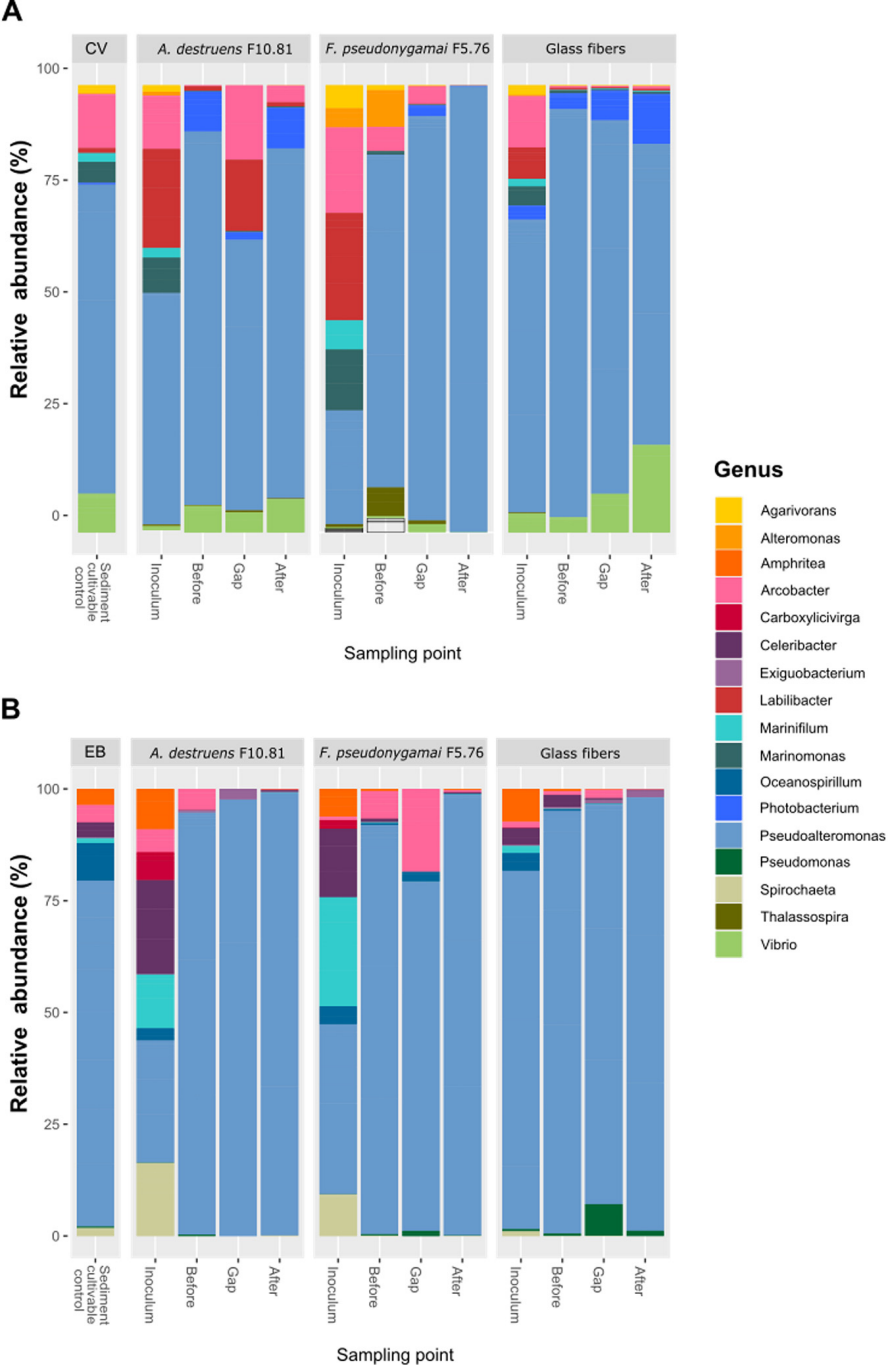
(A and B) Composition of the translocated bacterial communities obtained at the different sampling points with *A. destruens* F10.81, *F. pseudonygamai* F5.76, and glass fiber translocation networks from sediments from (A) Canal Vieil and (B) Etang de Berre. The analysis was performed at the genus level applying a threshold similarity of 97% for operational taxonomic unit (OTU) identification. Only genera with more than 0.1% relative abundance are presented. “Sediment” corresponds to the sediment cultivable control without a translocation network.

Consistently, the initial composition of the bacterial community (sediment cultivable control) from Canal Vieil sediment was different (PERMANOVA, *P* < 0.001) from that of Etang de Berre (Fig. 3). The cultivable bacterial community from Canal Vieil sediment was dominated by *Pseudoalteromonas*, *Arcobacter*, *Vibrio*, and *Marinomonas* (representing more than 93% ± 2%), while that from Etang de Berre was dominated by *Pseudoalteromonas*, *Arcobacter*, *Oceanospirilum*, *Amphritea*, and *Celeribacter* (representing more than 94% ± 1.4%). Notably, except for *Amphritea* and *Celeribacter*, the members of all the other genera have been described as facultative anaerobes (58). The presence of the fungi modified the bacterial composition (PERMANOVA, *P* < 0.05) with increasing *Labilibacter* and *Arcobacter* abundances from Canal Vieil inoculum, while *Celeribacter*, *Spirochaeta*, and *Marinifilum* were more abundant in the inoculum from Etang de Berre (Fig. 3). However, the composition of the inoculum with glass fibers was unchanged (PERMANOVA, *P* > 0.05) in comparison to sediment cultivable controls. This observation suggested that the presence of fungi creates specific conditions favoring the growth of such microorganisms under the experimental conditions. It is important to note that most of these dominant genera are known to be motile (58), which is a feature that has been shown to be crucial for hyphosphere colonization (57). In addition, these genera probably have the capacity to resist to PAHs and grow fast in the cultured media used, facilitating colonization. Indeed, *Amphritea*, *Arcobacter*, *Celeribacter*, *Marinifilum*, *Marinomonas*, *Oceanospirillum*, *Pseudoalteromonas*, *Spirochaeta*, and *Vibrio* are known to adopt a K-strategy, growing fast in the presence of hydrocarbons (59–65).

The bacterial composition was modified in the sampling points (before, in the gap, and after) along the translocation networks, in agreement with nonmetric multidimensional scaling (NMDS). As a result, *Pseudoalteromonas* was found to be dominant after the gap for all translocation networks. Obviously, *Pseudoalteromonas* was the most adapted to the experimental conditions, allowing it to fully cover the hyphosphere. Nevertheless, different genera were observed according to the translocation networks represented at low abundance after the gap (Fig. 3), which might represent the selection of specific genera related to FBI, consistent with alpha diversity observations.

### Identification of ASVs selected during translocation.

In order to identify the ASVs specifically associated with a translocation network the linear discriminant analysis effect size (LEfSe) was performed considering the bacterial community from both sediments after the gap. LEfSe revealed statistically significantly more abundant ASVs for a translocation network. Such ASVs, more abundant according to the condition, have been proposed as biomarkers (66), which likely correspond in our study to selected ASVs by fungi.

**FIG 4:**
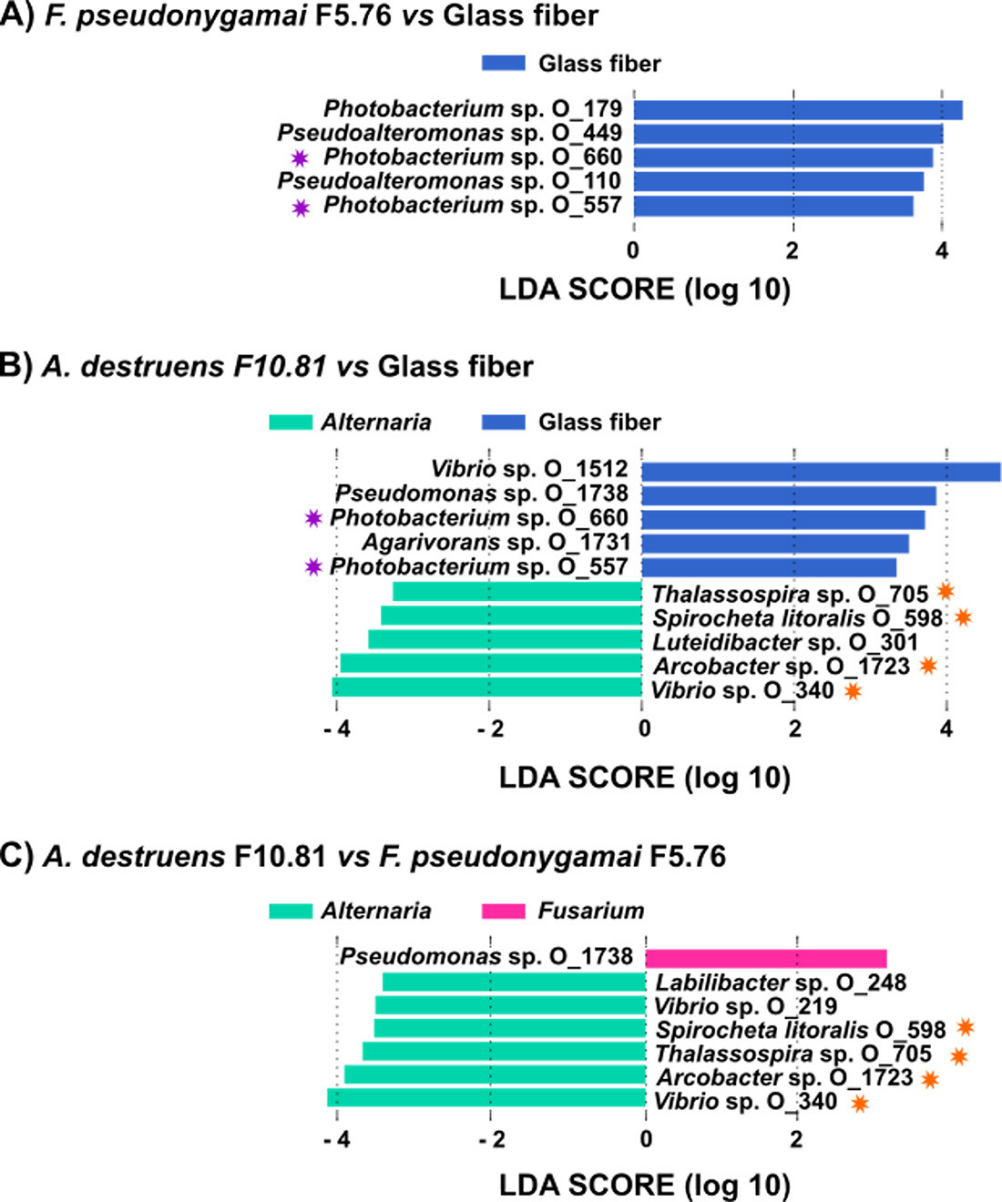
Comparison of translocated bacterial communities according to the translocation network. (A to C) The linear discriminant analysis effect size (LEfSe) identified significant differential abundance distribution of bacterial ASVs, from bacterial communities obtained after the gap, between *A. destruens* F10.81 hyphae and glass fibers (A), between *F. pseudonygamai* F5.76 hyphae and glass fibers (B), and between both fungi (C). The analysis was performed considering bacterial communities obtained after the gap with both Etang de Berre and Canal Vieil sediments. The stars indicate ASV biomarkers identified for *A. destruens* F10.81(orange) and glass fibers (purple) present in the two comparisons performed for each translocation network.

When comparing fungal translocation networks with the glass fibers network, eight ASVs significantly more abundant with glass fibers were identified, while five ASVs were significantly more abundant with *A. destruens* F10.81 (Fig. 4A and B). When comparing *A. destruens* F10.81 and *F. pseudonygamai* F5.76 translocation networks, one ASV was found to be significantly more abundant with *F. pseudonygamai* F5.76 (ASV affiliated to Pseudomonas spp.) and six ASVs significantly more abundant with *A. destruens* F10.81 (Fig. 4C). The different numbers of ASVs significantly more abundant for each fungal translocation network can be explained by the fact that the fungi have different cell surface properties as evidenced by different PAH uptake strategies (42), even if other parameters cannot be excluded. Interestingly four ASVs identified as specifically more abundant for *A. destruens* F10.81 were found common compared with either glass fibers (Fig. 4B) or *F. pseudonygamai* F5.76 (Fig. 4C). Such an observation suggested that these ASVs have been specifically selected along the *A. destruens* F10.81 translocation network and can therefore be considered ASVs able to establish a specific link with *A. destruens* F10.81. These selected ASVs are related to *Thalassospira* spp., Spirochaeta litoralis, *Arcobacter* spp., and *Vibrio* spp. It has been described that fungi produce small signaling molecules (67), including chemo-attractive compounds (68), resulting in the selection of bacteria able to be translocated. Also, antibiotic-based mechanisms have been described for the selection of insensitive or antibiotic-resistant bacteria by fungi (19). All the selected ASVs are affiliated with a genus detected in marine sediments in the presence of hydrocarbons (60, 69–72). To the best of our knowledge, these genera have not been described as associated with *Alternaria* spp. It was surprising that Spirochaeta litoralis was selected by the *A. destruens* F10.81 translocation network under our experimental aerobic conditions because it is known as a strict anaerobic bacterium (73, 74). Such an observation suggested that anaerobic microniches were created along the fungal translocation network development.

**FIG 5:**
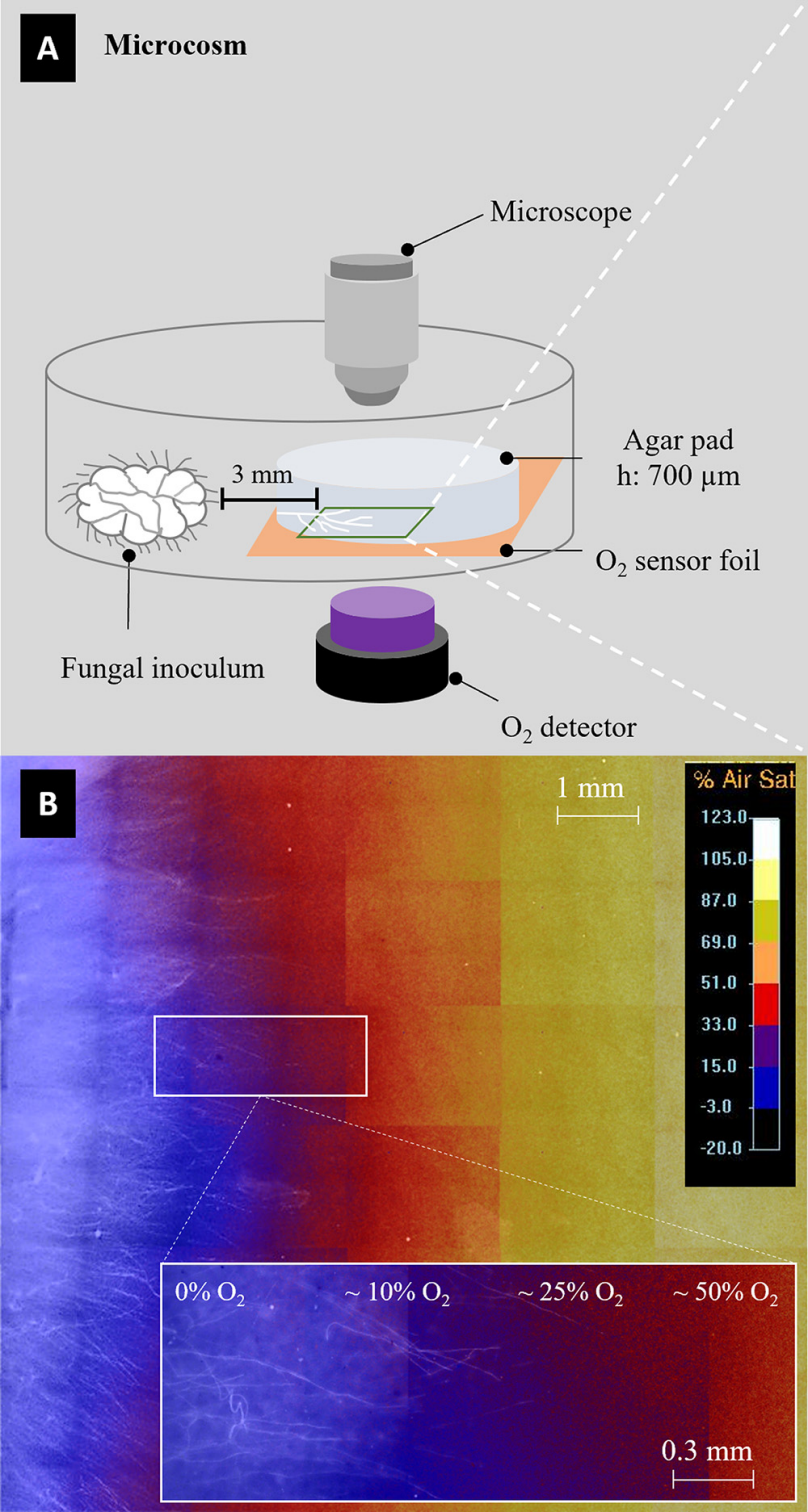
Oxygen profiles during fungal growth. (A) Schematic view of the microcosm used for mycosphere oxygen mapping; (B) spatially distinct oxygen gradient (0 to 100%) observed in the mycosphere of *A. destruens* F10.81. The anoxic environment (indicated by blue color) was observed in the mycosphere where a dense hyphal network was established.

To further explore this hypothesis, the oxygen concentration was measured in the hyphosphere during the development of the fungal mycelia. Oxygen depletion was observed during the mycelial growth (Fig. 5), showing that favorable conditions are created for the translocation of anaerobic bacteria along the fungal network. Such an observation further supports that fungi establish specific links with anaerobic bacteria (45). It is likely that fungi that create anaerobic niches have a crucial role in the organization of microbial communities, playing a key role in the dispersal of anaerobic bacteria (75). Using time-resolved optical oxygen mapping, microscopy, and metabolite analysis, Xiong et al., for instance, revealed fungal growth-induced formation and persistence of anoxic circum hyphal niches that allowed for spore germination, dispersal, and growth of the obligate anaerobe Clostridium acetobutylicum along hyphae of the litter-decaying fungus Coprinopsis cinerea (75). Thus, anaerobic conditions created by the fungal growth might allow anaerobic bacteria to survive at the aerobic/anaerobic interface and to overcome hydrocarbon hydrophobic patches in PAH-contaminated sediments. Such fungal translocation has been demonstrated for bacterial dispersion and colonization of different niches (10, 26). A similar anoxic condition (84 to 96 h, data not shown) was also observed in the hyphosphere of the fungus *F. pseudonygamai* F5.76. Under given experimental conditions and likely due to different metabolic activity, this fungal strain, however, formed anoxic hyphosphere conditions ca. 28 h later than *A. destruens* F10.81 (data not shown). The late occurrence of the anoxic microniche may explain the absence of anaerobic bacteria along the *F. pseudonygamai* F5.76 transportation networks in our experimental system. Overall, our results not only confirm our hypothesis that fungi select bacteria to be translocated through fungal mycelia but also show that the same fungus selects aerobic and anaerobic bacteria. We propose that aerobic bacteria are transported in the oxygen-rich apical hyphae zone, while anaerobic bacteria follow after oxygen depletion by fungal growth. However, further research is required to characterize and understand the translocation of anaerobic bacteria by fungal mycelia.

**Conclusion.** Despite the fact that our experimental conditions selected the fast-growing bacteria *Pseudoalteromonas*, our study demonstrated that the fungal translocation network selected specific bacterial populations, especially for *A. destruens* F10.81, while *F. pseudonygamai* F5.76 was not able to select specific bacteria. The cell surface properties likely explain the behavior of fungi, as they showed different PAH uptake mechanisms. The PAH uptake by *A. destruens* F10.81 results in a homogenous distribution of PAH into the fungal cells that probably relies on a diffusion mechanism. The cell surface likely has hydrophobic properties that would be beneficial for the interaction with and the selection of bacteria, especially in the presence of PAH. Further studies are required to elucidate the mechanism involved in bacterial selection. Among the four identified bacteria specifically selected by *A. destruens* F10.81, it was striking to observe the presence of anaerobic bacteria (Spirochaeta litoralis). Such a result suggested that the oxygen depletion during the hyphal development created anoxic niches, allowing facultative and anaerobic bacteria to be dispersed thanks to the fungal translocation network. Our results also show that the selected bacteria can be translocated through hydrophobic patches (hydrocarbon-contaminated hydrophobic sediment gap) via the fungal network. Such a result is of environmental relevance, indicating that anaerobic and aerobic bacteria can be dispersed, particularly translocated through different hydrophobic patches via the fungal network, for the colonization of novel niches. The microorganisms involved in our experiment are from hydrocarbon-contaminated coastal sediment, indicating that fungi and bacteria from the benthic compartment are able to specifically interact. The experiment in petri dishes is far from representative of coastal sediment conditions; therefore, further studies, under sediment conditions, are required to elucidate the fungal-bacterial interaction (FBI) in coastal sediment. Because most microorganisms have a lifestyle involving microbial interactions (biofilm attached to particles), FBI deserves further study in coastal sediments.

## MATERIALS AND METHODS

### Culture medium.

To mimic marine conditions, seawater minimal medium (swMM) was used with the following composition: KCl, 0.75 g L^−1^; CaCl · 2H_2_O, 1.47 g L^−1^; NH_4_Cl, 1.5 g L^−1^; MgSO_4_ · 7H_2_O, 6.64 g L^−1^; NaCl, 20 g L^−1^; Na_2_CO_3_, 0.265 g L^−1^; 1 mL of trace element solution [H_3_BO_3_, 300 mg L^−1^; FeSO_4_ · 7H_2_O, 1.1 g L^−1^; CoCl_2_ · 6H_2_O, 190 mg L^−1^; MnCl_2_ · 2H_2_O, 50 mg/L; ZnCl_2_, 42 mg/L; NiCl_2_ · 6H_2_O, 24 mg/L; Na_2_MoO_4_ · 2H_2_O, 2 mg L^−1^], 1 mL of vitamin solution (biotin, 2 mg L^−1^; ρ-aminobenzoate, 10 mg L^−1^; thiamine, 10 mg L^−1^; pantothenate, 5 mg L^−1^; pyridoxamine, 50 mg L^−1^; vitamin B_12_, 20 mg L^−1^; nicotinate, 20 mg L^−1^), and 100 μL of phosphate buffer 50 mM. The pH was adjusted with HCl to 6.5. Chemicals were purchased from Sigma-Aldrich (Germany).

### Fungal strains.

Two fungi, *Alternaria destruens* F10.81 and Fusarium pseudonygamai F5.76, exhibiting different PAH uptake strategies, were isolated from a heavily PAH-contaminated coastal sediment (Etang de Berre, France) (42). *A. destruens* F10.81 has the capacity to internalize homogenous PAH, while *F. pseudonygamai* F5.76 forms PAH vacuoles. Fungi were maintained in malt dextrose agar (MDA) supplemented with swMM to maintain salinity conditions (MDAsw). Their capacity to translocate bacteria was tested in swMM with 10% Luria Bertani agar medium in swMM (swLB) medium containing 30 mg L^−1^ of pyrene and 20 g L^−1^ agar, inoculated with sediments. Microscopic observations were performed with a microscope (Eclipse E600, Nikon, Kawasaki, Japan) at ×100 magnification.

### Bacteria isolation.

Sediments from Etang de Berre (France, 43°2905″N; 5°11′’17″E), having high hydrocarbon content (around 280 μg g^−1^) (70), were inoculated directly in Luria-Bertani (LB) medium prepared with swMM (LBsw) to maintain the salinity. After 48 h of growth at room temperature (20°C), eight phenotypically different bacterial colonies were observed. A representative of each phenotype was randomly selected and reinoculated in separated plates of LBsw medium until pure cultures were obtained. Bacteria were evaluated for swimming and swarming motility following standard motility test procedures as previously described (10). The capacity of bacteria to grow in 30 mg L^−1^ of pyrene as the only carbon source was tested in liquid swMM. DNA from bacteria was extracted from isolated colonies in swLB medium using the Qiagen DNeasy UltraClean microbial kit (catalog [cat.] no. 12224-40) following the manufacturer’s instructions. Bacteria were identified by determining the sequence of the 16S rRNA gene amplified by PCR using the forward primer 63f (5′-CAG GCC TAA CAC ATG CAA GTC-3′) and reverse primer 1387r (5′-GGG CGG WGT GTA CAA GGC-3′) (76). The PCR mix was prepared with 1 μL of extracted DNA in 9.5 μL of diethyl pyrocarbonate (DEPC)-treated water, 1 μL of each primer (20 μM), and 12.5 μL of AmpliTaq Gold 360 master mix 2× (Thermo Fisher Scientific, USA). The amplification was performed through 35 cycles of 95°C (45 s), 58°C (45 s), and 72°C (1 min), with a previous activation start of 95°C (10 min) and a final extension step at 72°C (10 min). Amplified fragments, with an approximate length of 1,300 bp, were sequenced at the Eurofins platform (France). Sequence data were edited using Chromas Pro version 1.34. For identification, 16S rRNA gene sequences were compared with the NCBI (National Centre for Biotechnology Information; http://www.ncbi.nlm.nih.gov) database. The obtained sequences have the NCBI database accession numbers OK128316 to OK128323.

### Bacterial dispersal experiments.

The bacterial dispersal experiments were set up in petri dishes (Ø: 5 cm) on swMM with 10% swLB medium containing 30 mg L^−1^ pyrene for maintaining PAH selective pressure and 20 g L^−1^ agar. A 2-mm separation gap was created by cutting the solidified medium, resulting in small (1.4 cm) and big (3.4 cm) compartments (Fig. 1A-a). The gaps were filled either with sterile wet sand, sterile sediment, or wax or left empty (emulating air pores) to mimic spatially distinct barriers as in heterogenic contaminated sediment (Fig. 1B). The barriers were chosen due to the differences in hydrophobic and hydrophilic characteristics. The petri dishes were inoculated at 3 mm from the border of the bigger compartment with actively growing mycelia cultivated in potato dextrose agar (PDA) medium. The petri dishes were then incubated at room temperature (20°C) in darkness until the hyphae grew to ~1 cm long (48 h) (Fig. 1A-b). Then, either single bacteria or sediments were inoculated in order to observe bacterial translocation. The single bacterial inoculation was used as a positive control to validate the effectiveness of bacterial translocation.

For the positive controls, the eight isolated bacteria (pregrown for 24 h in liquid swLB containing 30 mg L^−1^ pyrene) were inoculated close to the growing mycelia (Fig. 1A-c). The petri dishes were incubated in the dark at room temperature (20°C) for 10 days until the mycelia grew beyond the barriers (Fig. 1A-d). Bacterial translocation along hyphae was examined microscopically. Additional negative controls were prepared by inoculating the petri dishes with the previously mentioned eight bacteria without the presence of mycelia. All experiments were carried out in triplicate.

For the analysis of bacterial communities transported by a translocation network, the petri dishes were inoculated with sediments. In order to determine if the origin of the bacterial community influences the selection by fungi, sediments from two locations on the French Mediterranean coast were used: Etang de Berre (43°29′05″N; 5°11′17″E, hydrocarbon-polluted brackish sediment) and Canal Vieil cove (43°23′20″N; 4°59′31″E, hydrocarbon-polluted marine sediment), both showing hydrocarbon content around 280 μg g^−1 ^dry sediment (70, 77). The experiment for bacterial community translocation was designed in order to avoid fungal growth from the sediment inoculum: (i) the fungal translocation network (from *A. destruens* F10.81 or *F. pseudonygamai* F5.76) in the petri dish developed large enough hyphae before incubation by the sediments, and (ii) the incubation time for bacterial translocation after incubation of sediments was reduced to 10 days, avoiding having fungi present in the sediment inoculum develop hypha, as checked in the abiotic and negative controls. Controls were performed using glass fibers (Mühlmeier composite, Bärnau, Germany) as abiotic translocation networks and without a translocation network as the sediment cultivable control for bacteria and negative control for fungal growth, respectively. The samplings were performed at four points: in the inoculum, after the inoculum, in the gap (filled with a mixture of sediments from Etang de Berre and Canal Vieil sterilized by autoclaving), and after the gap (Fig. 1C). Fungal growth and bacterial colonies were observed after 10 days of incubation (Fig. 1D), and samples were placed in independent Eppendorf tubes. All experiments were performed in triplicate.

### DNA extraction and Illumina MiSeq sequencing.

DNA from the setups at each sampling point and from samples of sediments from Etang de Berre and Canal Vieil were extracted with a PowerSoil DNA isolation kit (MoBio Laboratories) according to the manufacturer’s recommendations. The V3 to V4 hypervariable region of the 16S rRNA gene was amplified using the bacteria primers 344F-V3V4 (5′-ACGGRAGGCAGCAG-3′) and 801R-V3V4 (5′-TACCAGGGTATCTAATCCT-3′). The reverse and forward primers included Illumina adapters. Before amplification, the concentration of DNA was determined with a Quant-It kit in a microplate in order to normalize the following amplification. Amplifications were performed in triplicate using AmpliTaq Gold 360 master mix (Applied Biosystems) to prepare 25-μL reactions containing 2 ng of DNA of bacteria. Cycling conditions were as follows: an initial denaturation step at 95°C for 10 min, followed by 35 cycles with a denaturation step at 95°C for 30 s, a hybridization step at 60°C for 30 s, and an elongation step at 72°C for 40 s or 30 s, after which a final elongation step at 72°C for 10 min was performed. Sequencing by synthesis was carried out at the Genotoul platform (Toulouse, France) with Illumina MiSeq. The 2 × 250-paired-end sequencing gave a full length of ~460 bp for the V3 to V4 region.

### Sequence processing.

The open-source software QIIME 2 (43) was used for processing 16S rRNA gene sequences. Sequencing resulted in 2,985,157 raw reads. The data set was first demultiplexed, and then DADA2 (78) was used to control the read quality and infer amplicon sequence variants (ASVs) (79). Default settings were used except that reverse reads were truncated to 230 bp before merging. This denoising step resulted in a 1,699,056 trimmed-sequence data set. The taxonomy was assigned to the ASVs with a fixed sequence similarity threshold of 97% using the SILVA database (80) release 132 for 16S rRNA gene sequences. Then, the data set was filtered to remove mitochondria, chloroplasts, and unassigned (at the domain level) sequences. Bioinformatics treatment resulted in 1,882 prokaryotic ASVs. Phylogenetic trees were constructed using FastTree (81) in the QIIME2 phylogeny plugin. The phylogenetic tree was made ultrametric for the following analyses using the Chronos function in the R package ape (82), with branch lengths representing relative time and all tips equidistant from the root. The data set encompassed 84 samples.

### Statistics.

Statistical analysis of the ASV richness of each sample and the Shannon diversity index were calculated comparing translocation networks and sampling points in R software version 4.0.3 (http://www.r-project.org/). Unconstrained nonmetric multidimensional scaling (NMDS) for sampling points and translocation networks was performed using Bray-Curtis distances in the metaMDS function (k = 3) of the vegan package 2.5-66. Differences of translocation network effects on the microbial communities were tested using permutational multivariate analysis of variance (PERMANOVA) with 9,999 permutations. Statistical differences in relative abundance of phyla between same groups were determined using a two-sample, two-tailed homoscedastic *t* test. To identify the significant abundant bacterial ASVs from both sediments between translocation networks after the gap, linear discriminant analysis effect size (LEfSe) analysis was applied; the analysis was run in Hutlab Galaxy (https://huttenhower.sph.harvard.edu/galaxy/).

### *In vivo* mapping of oxygen concentration in the mycosphere.

A plannar oxygen optode (SF-RPSu4, PreSens, Regensburg, Germany) and a VisiSens TD detector unit DU02 (PreSens) were used for time-lapse optical mapping of the oxygen concentration in the mycosphere of *A. destruens* F10.81 and *F. pseudonygamai* F5.76. A sensor foil (1 by 1 cm) was glued to the glass bottom of a petri dish (μ-Dish, 35 mm, low; Ibidi, Gräfelfing, Germany) using silicon glue. The petri dish was kept in the dark to let the silicon glue cure overnight. Then a circular agarose pad (Ø: 18 mm; height [h]: 0.7 mm, swMM plus 10% swLB medium plus 30 mg L^−1^ pyrene) was prepared as previously described (83) and placed on top of the sensor foil. A circular agarose pad (Ø: 10 mm) with the fungal inoculum was then placed at 1-mm distance from the oxygen-sensing pad. An observation area (1.8 by 2.5 mm, 4 mm from the fungal inoculum) was chosen in the oxygen-monitoring pad to monitor oxygen dynamics during fungal colonization. Specifically, images of the observation area were taken by the detector unit (exposure: 100,000 μm, gain: 10) with 1-h imaging intervals during the 96-h incubation. Time-lapse oxygen mapping experiments were performed in triplicate each for *A. destruens* F10.81 and *F. pseudonygamai* F5.76. Another, identical setup was used to map the spatial oxygen and mycelial distribution of the fungus *A. destruens* F10.81 after 48 h of incubation of the whole oxygen-monitoring area (1 by 1 cm). To screen for oxygen in the 1 by 1-cm sensor foil, 30 spots (cf. 1.8 by 2.5 mm per spot) were imaged with the VisiSens TD detector unit as follows: the position of the VisiSens TD detector unit was fixed while the position of the petri dish was precisely shifted using a microscope stage (Nikon AZ100, Tokyo, Japan) controlled by the microscope imaging software (large image acquisition, NIS-Elements, Basic Research, Nikon). Then, microscopic images were immediately taken to visualize mycelial distribution in the direct vicinity of the sensor foil using the Nikon microscope (AZ100, Tokyo, Japan). Briefly, bright-field images were taken with the Nikon microscope with a 5× lens objective and ×3 microscope magnification (a total magnification of ×15, 1.78 by 2.22 mm per image) with LED illumination (exposure time of 30 ms). Images taken with the VisiSens TD detector unit (spatial O_2_ information) and Nikon microscope (mycelial distribution) were grouped separately, and then the size of the two grouped images was standardized to 1.87 μm per pixel using ImageJ. The standardized images were then overlaid in ImageJ with 50% opacity to combine the spatial oxygen information and the mycelial distribution in the microcosm.

### Data availability.

The complete data set was deposited in the NCBI Sequence Read Archive (SRA) database (SUB10342502) and is available under the BioProject ID PRJNA761592.
